# Correction: Considerations in Platinum and Gold Drug Design and the Synthesis of Chloro-(2,3-Diphenyl-1,3,4-Thiadiazolium M-5-Thiolato-S_exo_)Gold(1): The First Gold-Mesoionic Complex of its Kind

**DOI:** 10.1155/MBD.1999.194

**Published:** 1999

**Authors:** P. F. de Athayde Filho, B. P. Howe, J. Miller

**Affiliations:** 1 Universidade Federal da Paraíba Departamento de Química CCEN, Campus I João Pessoa Paraíba CEP 58.51-970 Brazil; 2 Laboratorio de Tecnologia Farmaceutica-CCS Universidade Federal de Paraiba Cidade Universitaria Campus I Caixa Postal 5009 João Pessoa PB 58.051-970 Brazil


					CORRECTION
Metal-Based Drugs 4 (1997), 273
CONSIDERATIONS IN PLATINUM AND GOLD DRUG DESIGN
THE SYNTHESIS OF CHLORO-(2,3-DIPHENYL-1,3,4-
THIADIAZOLIUM-5-THIOLATO-Sexo)GOLD(I):
THE FIRST GOLD-MESOIONIC COMPLEX OF ITS KIND
AND
B. P. Howe
Universidade Federal da Parafba, Departamento de Qufmica, CCEN, Campus I,
JoSo Pessoa, Parafba, Brazil, CEP 58.51-970. E-mail: brianh@vm.npd.ufpb.br
The authors of this paper, published in Metal-Based Drugs 4 (1997), 273, should
be in fact
P. F. de Athayde Filho, B. P. Howe, and J. Miller*
Laboratorio de Tecnologia Farmaceutica-CCS, Universidade Federal de Paraiba
Cidade Universitaria, Campus I, Caixa Postal 5009
58.051-970 Joao Pessoa, PB, Brazil. E-mail: <millerjo@ltf.ufpb.br>
194

				

